# Supplementary material for: Prediction of Mutations and Outcome in Gastrointestinal Stromal Tumors with Deep Learning: A Multicenter, Multinational Study
Source: medRxiv. 2026 Feb 3:2026.02.02.26345350. Preprint. [Version 1] doi: 10.64898/2026.02.02.26345350 (PMC12889797; doi:10.64898/2026.02.02.26345350)
Supplement: 1 [file NIHPP2026.02.02.26345350V1-supplement-1.pdf]

medRxiv preprint doi: https://doi.org/10.64898/2026.02.02.26345350; this version posted February 3, 2026. The copyright holder for this preprint (which was not certified by peer review) is the author/funder, who has granted medRxiv a license to display the preprint in perpetuity.
It is made available under a CC-BY 4.0 International license .

Supplementary Methods

For the analysis of RFS, the DIGIST cohort was partitioned using the same geographical split and the same ratio for defining training, internal and external validation cohorts as for the molecular prediction model. (Supplementary Figure 1 A).

Different DL models were trained on C1, C2 and C3 training sets to address the prognostic tasks and were subsequently named DL scores C1, C2 and C3. For each cohort, a same pipeline was employed to evaluate the prognostic value of the scores and their added value compared to the mitotic count alone and combined with the other established prognostic variables from the AFIP criteria (i.e., tumor size, location and rupture) (Supplementary Figure SF1 B). Age, sex, and mutational status (as well as adjuvant TKI therapy for the C1 cohort) were systematically included as baseline covariates in all models to account for their potential confounding effect and to ensure consistent risk adjustment across model comparisons. Overall, five models were systematically trained on training C1, training C2 and training C3 relying on the (i) DL score (named simple DL model), (ii) DL score combined with tumor size, location and rupture (named deep Miettinen-Joensuu model), (iii) Mitotic count (named mitotic model), (iv) AFIP criteria provided by the pathologists (named pathological Miettinen-Joensuu model), and (v) mitotic count combined with tumor size, location and rupture (named continuous Miettinen-Joensuu model). The performance of these DL-based models were systematically compared against the three other benchmark models providing insights into whether AI-driven approaches can enhance or complement existing prognostic tools.

Univariable survival analysis including log-rank tests and Cox regressions was first performed in each training cohort on the variables entered in the models.

Afterwards, the multivariable models were trained using the Cox proportional hazard ratio (HR) model in the training cohorts and applied on the internal and external validation cohorts. Multivariable HRs with their 95% CI were calculated in the training cohorts. The performances of the models were estimated using Harrell concordance index (C-index, which assesses the discriminative ability of the models) and integrated Brier score (IBS, combined with prediction error curves, which quantifies overall prediction accuracy and calibration over time) over the first 10 years following resection with 95% CIs. In the training cohorts, the model performances were evaluated using 5-fold cross-validation, with evaluation performed on the out-of-fold samples (Supplementary Figure 1 B).

medRxiv preprint doi: https://doi.org/10.64898/2026.02.02.26345350; this version posted February 3, 2026. The copyright holder for this preprint (which was not certified by peer review) is the author/funder, who has granted medRxiv a license to display the preprint in perpetuity.
It is made available under a CC-BY 4.0 International license .

Model performances were then compared in the internal and external validation cohorts using permutation tests, where predicted risks were randomly swapped (with 1000 random label exchanges) between models to generate the null distribution of differences in c-index and IBS. P-values were computed as the proportion of permuted differences exceeding the observed one. Kaplan-Meier curves for RFS in each patient group were drawn after dichotomizing the DL scores according to their median in the training cohorts.

Supplementary Figures

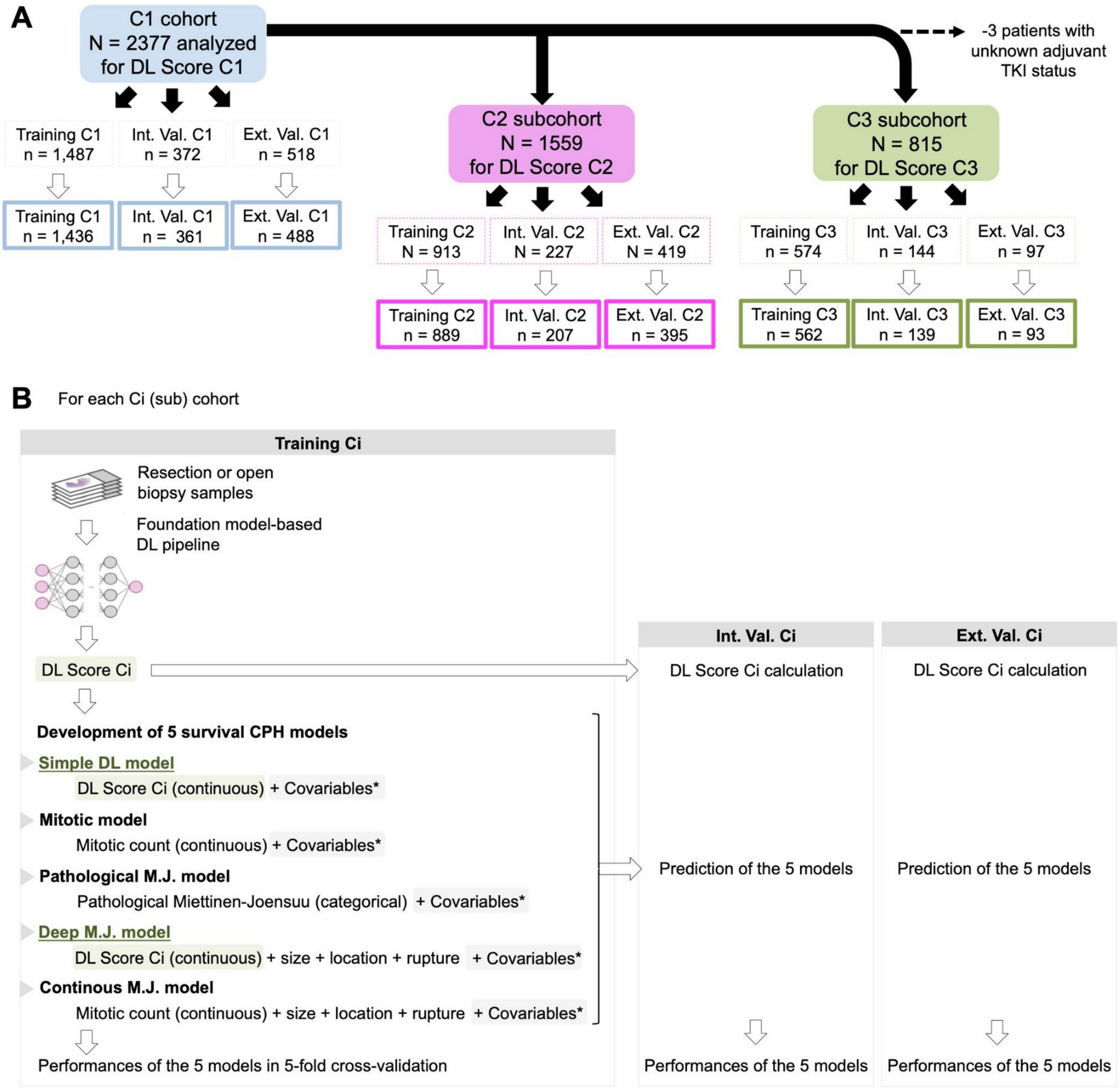

Supplementary Figure 1. Statistical pipeline for recurrence-free survival (RFS) analysis. (**A**) Data partitioning. Boxes with light dashed contours corresponded to the

medRxiv preprint doi: https://doi.org/10.64898/2026.02.02.26345350; this version posted February 3, 2026. The copyright holder for this preprint (which was not certified by peer review) is the author/funder, who has granted medRxiv a license to display the preprint in perpetuity.
It is made available under a CC-BY 4.0 International license .

patients with available DL Scores while boxes with solid thick contours to the patient included in the survival analysis. (**B**) Methodological approach performed in C1, C2 and C3 to obtain deep learning (DL) scores and to develop survival models in the Training cohorts using the Cox proportional hazard (CPH) algorithm and to compare them. Other abbreviations: ext.val: external validation, int. val: internal validation, M.J.: Miettinen-Joensuu. *Covariables were: age, sex, adjuvant TKI therapy and mutational status for C1; and age, sex and mutational status for C2 and C3.

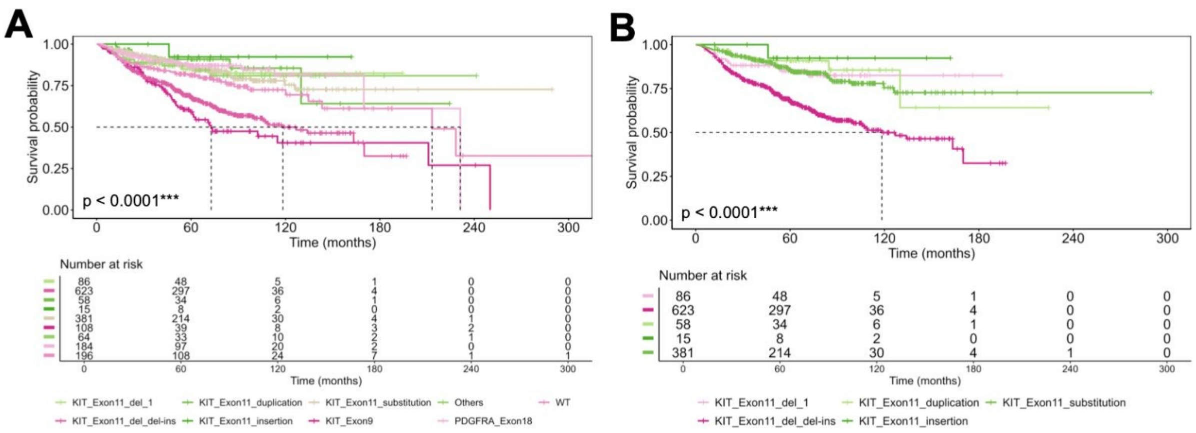

Supplementary Figure 2. **Associations between recurrence-free survival (RFS) and mutations.** Kaplan-Meier curves in the whole C1 cohort (unfiltered) for all mutations (**A**) and for KIT mutations (**B**) *: p < 0.05; **: p < 0.005; ***: p < 0.001. Tests are log-rank tests.

medRxiv preprint doi: https://doi.org/10.64898/2026.02.02.26345350; this version posted February 3, 2026. The copyright holder for this preprint (which was not certified by peer review) is the author/funder, who has granted medRxiv a license to display the preprint in perpetuity.
It is made available under a CC-BY 4.0 International license .

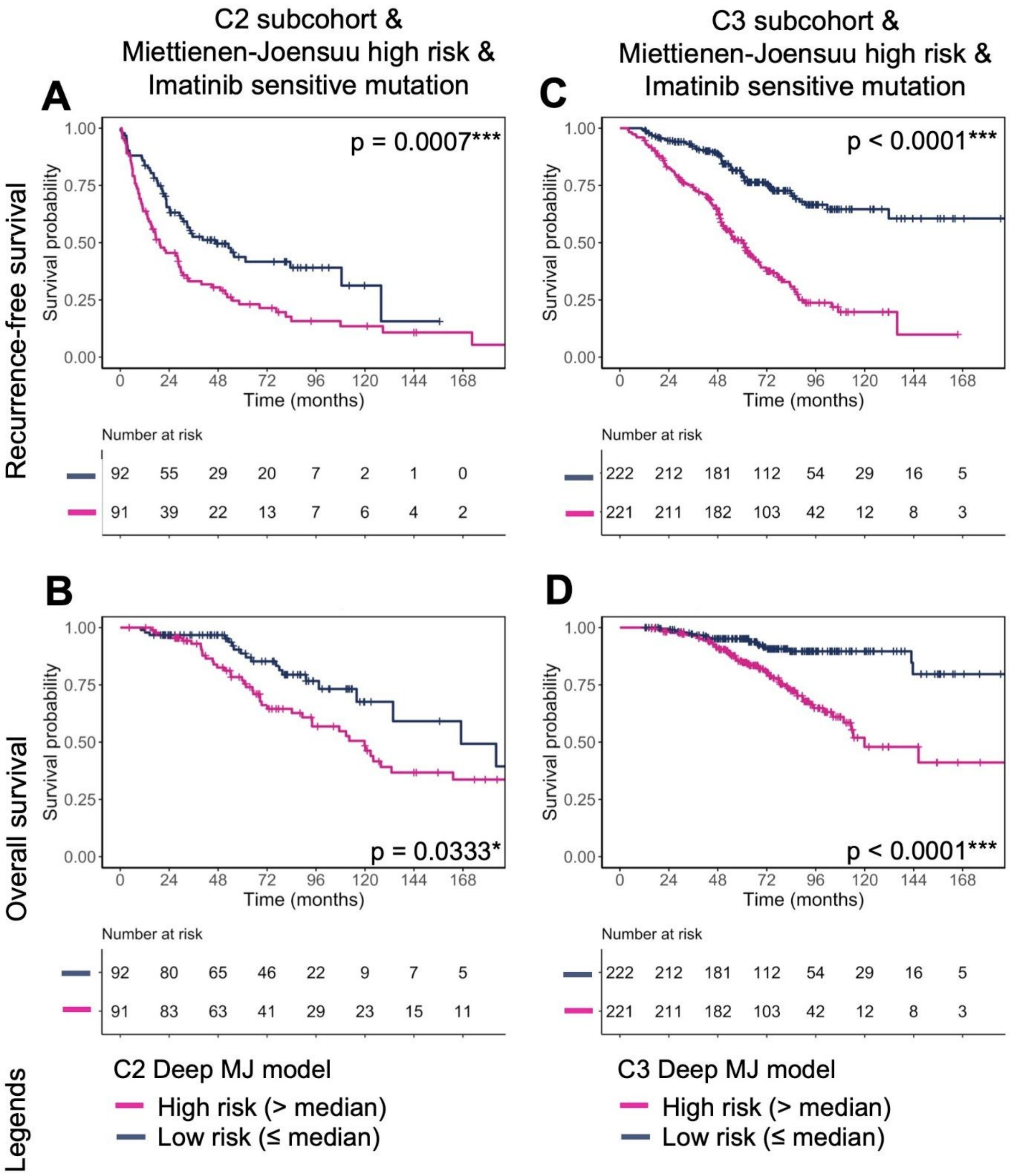

Supplementary Figure 3. **Kaplan-Meier curves for recurrence-free survival (RFS) and overall survival (OS) in the subgroup of C2 and C3 patients with high risk score according to pathological Miettienen-Joensuu scoring system and Imatinib sensitive mutations depending on the deep Miettinen-Joensuu models**. Kaplan-Meier curves for RFS (**A**) and OS (**B**) depending on the deep Miettinen-Joensuu model employing the C2 DL Score in this subgroup of the C2 subcohort. Kaplan-Meier curves for RFS (**C**) and OS (**D**) depending on the deep Miettinen-Joensuu model employing the C3 DL Score in this subgroup of the C3 subcohort. *: p < 0.05; ***: p < 0.001. Tests are log-rank tests.

medRxiv preprint doi: https://doi.org/10.64898/2026.02.02.26345350; this version posted February 3, 2026. The copyright holder for this preprint (which was not certified by peer review) is the author/funder, who has granted medRxiv a license to display the preprint in perpetuity.
It is made available under a CC-BY 4.0 International license .

Supplementary Tables

Country	Center	successfully processed WSI	Staining	Scanner	samples used with molecular mutation	samples with follow-up
Germany	Gerhard-Domagk-Institut für Pathologie UKM	1804	H&E	Nanozoomer S360 (Hamamatsu Photonics, Hamamatsu, Japan)	1732	32
	Universitätsmedizin Mannheim	142	H&E	3DHISTECH Pannoramic 480 digital scanner	34	126
France	Institut Bergonié	1825	HES	Nanozoomer S360 (Hamamatsu Photonics, Hamamatsu, Japan)	1796	348
	Ambroise Paré and University Hospital Reims	1385	HES	Nanozoomer S360 (Hamamatsu Photonics, Hamamatsu, Japan)	1375	79
	Centre Léon Bérard	540	HES	Aperio AT2 (Leica Biosystems, France)	475	264
	Gustave Roussy	457	HES	Aperio AT2 (Leica Biosystems)		184
Italy	Azienda Ospedale Università di Padova	180	H&E	Aperio LV1 (Leica Biosystems)	87	45
	IRCCS Istituto Nazionale dei Tumori	299	H&E	Aperio GT 450 DX (Leica Biosystems)	242	215
Netherlands	Netherlands Cancer Institute	179	H&E	3DHISTECH Pannoramic 1000 digital scanner	166	102
	Leiden University Medical Center	227	H&E		210	163
Japan	Japan STAR registry	475	H&E	NanoZoomer S360 (Hamamatsu Photonics,	473	467
	National Cancer Center Hospital	282	H&E		194	221

medRxiv preprint doi: https://doi.org/10.64898/2026.02.02.26345350; this version posted February 3, 2026. The copyright holder for this preprint (which was not certified by peer review) is the author/funder, who has granted medRxiv a license to display the preprint in perpetuity.
It is made available under a CC-BY 4.0 International license .

				Hamamatsu, Japan)		
**Poland**	Narodowy Instytut Onkologii im. Marii Skłodowskiej-Curie	217	H&E	NanoZoomer 2.0-RS (C10730 - Hamamatsu NanoZoomer 2.0-RS (C10730)	143	141
**Spain**	Hospital Oncológico en Valencia IVO	30	H&E	3DHISTECH Pannoramic 250 digital scanner	24	22
	La Fe University and Polytechnic Hospital Valencia	93	H&E	3DHISTECH Pannoramic 250 digital scanner	64	93
	Hospital Sant Pau Barcelona	46	H&E	3DHISTECH Pannoramic 250 digital scanner	44	-
	Hospital General Universitario Gregorio Marañón Madrid	57	H&E	3DHISTECH Pannoramic 250 digital scanner	-	56
	Hospital Germans Trias i Pujol Barcelona	59	H&E	3DHISTECH Pannoramic 250 digital scanner	59	48
	Hospital Clinic Valencia	55	H&E	3DHISTECH Pannoramic 250 digital scanner	45	
	Hospital Virgen del Rocio Sevilla	46	H&E	3DHISTECH Pannoramic 250 digital scanner	43	32
	Hospital Clinic Barcelona	111	H&E	3DHISTECH Pannoramic 250 digital scanner	32	-

Supplementary Table 1. Sample collection and processing across participating centers. For each center, the table reports (1) the number of successfully processed slides (defined as slides from which features were correctly extracted), (2) the number of samples per cohort with available molecular mutation data, and (3) the number of samples with complete information required for inclusion in the RFS prediction analysis.

medRxiv preprint doi: https://doi.org/10.64898/2026.02.02.26345350; this version posted February 3, 2026. The copyright holder for this preprint (which was not certified by peer review) is the author/funder, who has granted medRxiv a license to display the preprint in perpetuity.
It is made available under a CC-BY 4.0 International license .

<table>
<tr><th>column_name</th><th>column_class</th><th>uniqueness</th><th>requiredness</th><th>multiple_values</th><th>allowed_values</th></tr>
<tr><td>Country</td><td>character</td><td>non-unique</td><td>required</td><td>FALSE</td><td>Italy|Germany|Spain|France|Netherlands|Japan|</td></tr>
<tr><td>Center</td><td>character</td><td>non-unique</td><td>required</td><td>FALSE</td><td>(?:[a-z]|[A-Z])[a-zA-Z0-9]+</td></tr>
<tr><td>Patient_ID</td><td>character/integer</td><td>non-unique</td><td>optional</td><td>FALSE</td><td>[0-9a-zA-Z]\S+</td></tr>
<tr><td>Sample_ID</td><td>character/integer</td><td>unique</td><td>required</td><td>FALSE</td><td>[0-9a-zA-Z]\S+</td></tr>
<tr><td>Sex</td><td>character</td><td>non-unique</td><td>optional</td><td>FALSE</td><td>female|male</td></tr>
<tr><td>Date_of_Birth</td><td>date</td><td>non-unique</td><td>optional</td><td>FALSE</td><td>%m/%Y</td></tr>
<tr><td>Age_at_Diagnosis</td><td>integer</td><td>non-unique</td><td>optional</td><td>FALSE</td><td>[0-9]+</td></tr>
<tr><td>Last_news_date</td><td>date</td><td>non-unique</td><td>required</td><td>FALSE</td><td>%m/%Y</td></tr>
<tr><td>Last_News_status</td><td>character</td><td>non-unique</td><td>required</td><td>FALSE</td><td>dead|alive</td></tr>
<tr><td>Cause_of_death</td><td>character</td><td>non-unique</td><td>optional</td><td>FALSE</td><td>(?:[a-z]|[A-Z])[a-zA-Z0-9]+</td></tr>
<tr><td>Date_of_death</td><td>date</td><td>non-unique</td><td>optional</td><td>FALSE</td><td>%m/%Y</td></tr>
<tr><td>Significant_Previous_History</td><td>character</td><td>non-unique</td><td>optional</td><td>TRUE</td><td>yes|no|NF1|Stratakis-Carney Dyad|Carney Triad|Familial GIST</td></tr>
<tr><td>Initial_Diagnosis_date</td><td>date</td><td>non-unique</td><td>optional</td><td>FALSE</td><td>%m/%Y</td></tr>
<tr><td>Pathology_Laboratory</td><td>character</td><td>non-unique</td><td>optional</td><td>FALSE</td><td>(?:[a-z]|[A-Z])[a-zA-Z0-9]+</td></tr>
<tr><td>Type_of_Sampling</td><td>character</td><td>non-unique</td><td>optional</td><td>FALSE</td><td>resection|core_needle_biopsy|open_biopsy|FNA|TMA|endoscopic biopsy|Microbiopsy</td></tr>
<tr><td>Date_of_Sampling</td><td>date</td><td>non-unique</td><td>required</td><td>FALSE</td><td>%m/%Y</td></tr>
<tr><td>Site_of_Tumor</td><td>character</td><td>non-unique</td><td>optional</td><td>TRUE</td><td>gastric|small_intestine|colorectal|other</td></tr>
<tr><td>Size_of_Tumor_mm</td><td>integer</td><td>non-unique</td><td>optional</td><td>FALSE</td><td>[0-9]+</td></tr>
</table>

medRxiv preprint doi: https://doi.org/10.64898/2026.02.02.26345350; this version posted February 3, 2026. The copyright holder for this preprint (which was not certified by peer review) is the author/funder, who has granted medRxiv a license to display the preprint in perpetuity.
It is made available under a CC-BY 4.0 International license .

<table>
<tr><td>Mitotic_index_5mm2</td><td>integer</td><td>non-unique</td><td>optional</td><td>FALSE</td><td>[0-9]+</td></tr>
<tr><td>Mutation_Nucleotidic_Formula</td><td>character</td><td>non-unique</td><td>optional</td><td>FALSE</td><td>c\.\d+(_\d+)?[a-zA-Z]+(>[a-zA-Z]+)?</td></tr>
<tr><td>Mutation_Proteic_Formula</td><td>character</td><td>non-unique</td><td>optional</td><td>FALSE</td><td>.*</td></tr>
<tr><td>Mutation_Status_1</td><td>character</td><td>non-unique</td><td>required</td><td>FALSE</td><td>(KIT_Exon(12|14|8|5|11|13|17|9)|KIT_OtherExon|PDGFRA_Exon(10|11|12|14|18)|PDGFRA_OtherExon|WT for KIT-PDGFRA|Not Done|WT)</td></tr>
<tr><td>Mutation_Status_2</td><td>character</td><td>non-unique</td><td>optional</td><td>FALSE</td><td>.*</td></tr>
<tr><td>DOG1</td><td>character</td><td>non-unique</td><td>optional</td><td>FALSE</td><td>positive|negative</td></tr>
<tr><td>CD117</td><td>character</td><td>non-unique</td><td>optional</td><td>FALSE</td><td>positive|negative</td></tr>
<tr><td>Date_of_Surgery</td><td>date</td><td>non-unique</td><td>optional</td><td>FALSE</td><td>%m/%Y</td></tr>
<tr><td>Tumour_rupture</td><td>character</td><td>non-unique</td><td>optional</td><td>FALSE</td><td>yes|no</td></tr>
<tr><td>Adjuvant_TKI</td><td>character</td><td>non-unique</td><td>required</td><td>FALSE</td><td>yes|no</td></tr>
<tr><td>Duration_of_Adjuvant_TKI_months</td><td>integer</td><td>non-unique</td><td>required</td><td>FALSE</td><td>[0-9]+</td></tr>
<tr><td>Recurrence</td><td>character</td><td>non-unique</td><td>required</td><td>FALSE</td><td>yes|no</td></tr>
<tr><td>Date_of_recurrence</td><td>date</td><td>non-unique</td><td>required</td><td>FALSE</td><td>%m/%Y</td></tr>
<tr><td>Risk_stratification</td><td>character</td><td>non-unique</td><td>required</td><td>FALSE</td><td>high|low|intermediate</td></tr>
<tr><td>Miettinen_AFIP</td><td>character</td><td>non-unique</td><td>required</td><td>FALSE</td><td>very_low|low|moderate|high</td></tr>
</table>

Supplementary table 2 Template used for clinical data collection and harmonization across centers. The table defines all potential data fields included in the study, specifying the expected data type (*column_class*), uniqueness within the dataset, requiredness for analysis, allowance for multiple values, and permitted or regular-expression–defined entries.

medRxiv preprint doi: https://doi.org/10.64898/2026.02.02.26345350; this version posted February 3, 2026. The copyright holder for this preprint (which was not certified by peer review) is the author/funder, who has granted medRxiv a license to display the preprint in perpetuity.
It is made available under a CC-BY 4.0 International license .

This schema served as both a reference for data collection and as the grammar underlying the automated Grammar Data Curation Tool.

Molecular Mutation	Accuracy	Precision	Recall	F1	AUC	CI_lower	CI_upper
WT	0.78	0.21	0.41	0.27	0.64	0.58	0.68
KIT exon11	0.61	0.61	1.00	0.76	0.53	0.48	0.58
KIT exon9	0.62	0.17	0.90	0.28	0.80	0.76	0.84
PDGFRA exon18	0.68	0.29	0.77	0.42	0.71	0.66	0.75
other mutations	0.05	0.05	1.00	0.10	0.62	0.56	0.68
PDGFRA exon18 D842V	0.08	0.08	1.00	0.15	0.78	0.73	0.83
PDGFRA exon18 other	0.92	0.20	0.19	0.20	0.68	0.63	0.74
KIT del-inc557-558	0.70	0.18	0.37	0.24	0.55	0.48	0.61
KIT exon11 other mutation	0.45	0.43	0.95	0.59	0.53	0.47	0.59
KIT	0.73	0.73	1.00	0.84	0.56	0.51	0.61
PDGFRA	0.17	0.17	1.00	0.29	0.70	0.65	0.76
KIT 2del ins-del	0.73	0.38	0.42	0.40	0.64	0.59	0.70

Supplementary Table 3. Performance of ML models trained exclusively on clinical variables (sex, age, tumor location, mitotic index, and tumor size) for prediction of individual molecular mutations in GIST. For each mutation, the table reports accuracy, precision, recall, and the AUC with 95% CI.

Molecular Mutation	Test set	Fully Trained			Embedding and Mamba-2 frozen			Fully Frozen		
		AUC	CI_lower	CI_upper	AUC	CI_lower	CI_upper	AUC	CI_lower	CI_upper
KIT	internal validation	0.6	0.58	0.62	0.59	0.57	0.61	0.6	0.58	0.62
KIT	external validation	0.63	0.6	0.66	0.61	0.58	0.64	0.57	0.53	0.6
PDGFRA	internal validation	0.92	0.9	0.93	0.92	0.91	0.94	0.91	0.89	0.92
PDGFRA	external	0.93	0.89	0.95	0.93	0.91	0.95	0.93	0.9	0.95

medRxiv preprint doi: https://doi.org/10.64898/2026.02.02.26345350; this version posted February 3, 2026. The copyright holder for this preprint (which was not certified by peer review) is the author/funder, who has granted medRxiv a license to display the preprint in perpetuity.
It is made available under a CC-BY 4.0 International license .

	validation									
WT	internal validation	0.69	0.64	0.74	0.72	0.66	0.77	0.7	0.65	0.76
WT	external validation	0.61	0.52	0.68	0.66	0.57	0.73	0.62	0.53	0.71
KIT exon11	internal validation	0.59	0.57	0.61	0.59	0.57	0.61	0.59	0.57	0.61
KIT exon11	external validation	0.61	0.58	0.65	0.62	0.58	0.65	0.62	0.58	0.65
KIT exon9	internal validation	0.83	0.77	0.88	0.81	0.75	0.86	0.8	0.74	0.85
KIT exon9	external validation	0.63	0.56	0.71	0.66	0.57	0.74	0.67	0.57	0.75
PDGFRA exon18	internal validation	0.9	0.88	0.92	0.91	0.89	0.92	0.89	0.87	0.91
PDGFRA exon18	external validation	0.91	0.87	0.93	0.91	0.87	0.93	0.9	0.86	0.93
other mutations	internal validation	0.67	0.6	0.75	0.69	0.62	0.76	0.68	0.61	0.76
other mutations	external validation	0.57	0.43	0.68	0.63	0.51	0.73	0.62	0.5	0.73
PDGFRA Exon18 D842V	internal validation	0.88	0.85	0.91	0.89	0.85	0.92	0.87	0.83	0.9
PDGFRA Exon18 D842V	external validation	0.89	0.83	0.94	0.9	0.85	0.95	0.9	0.85	0.95
PDGFRA Exon18 other mutation	internal validation	0.91	0.88	0.94	0.91	0.87	0.95	0.91	0.87	0.94
PDGFRA Exon18 other mutation	external validation	0.87	0.81	0.92	0.85	0.79	0.9	0.85	0.78	0.91
KIT del-inc557-558	internal validation	0.78	0.75	0.81	0.77	0.74	0.8	0.76	0.73	0.79
KIT del-inc557-558	external validation	0.63	0.56	0.7	0.61	0.54	0.69	0.6	0.54	0.68
KIT Exon11 other mutation	internal validation	0.48	0.46	0.51	0.48	0.46	0.51	0.48	0.46	0.51
KIT Exon11 other mutation	external validation	0.51	0.46	0.55	0.53	0.48	0.57	0.52	0.47	0.57
KIT 2del ins-del	internal validation	0.81	0.78	0.83	0.81	0.79	0.83	0.8	0.78	0.82
KIT 2del ins-del	external validation	0.71	0.66	0.76	0.67	0.6	0.73	0.66	0.6	0.72

medRxiv preprint doi: https://doi.org/10.64898/2026.02.02.26345350; this version posted February 3, 2026. The copyright holder for this preprint (which was not certified by peer review) is the author/funder, who has granted medRxiv a license to display the preprint in perpetuity.
It is made available under a CC-BY 4.0 International license .

Supplementary Table 4. Predictive performance of the COBRA model for molecular mutations in GIST, reported as AUC with 95% CI for both the internal validation and the external validation. Results are presented for three training setups: fully trained, embedding and Mamba-2 layer frozen, and fully frozen.

Treatment	Mutations
**imatinib-sensitive**	KIT Exon 11
	KIT Exon 13 (V654A excluded)
	KIT Exon 17 (D816V, D820E and N822K excluded)
	PDGFRA Exon 12
	PDGFRA Exon 18 Non-D842V
**imatinib-dose-adjust**	KIT Exon 9
**avapritinib-sensitive**	PDGFRA Exon 18
	PDGFRA Exon 12

Supplementary Table 5. Grouping of mutations according to treatment sensitivity categories used for DL-based prediction. The table defines how specific *KIT* and *PDGFRA* mutations were classified as imatinib-sensitive, imatinib–dose-adjust, or avapritinib-sensitive

Treatment	Accuracy	Precision	Recall	F1	AUC	CI_lower	CI_upper
imatinib-sensitive	0.65	0.65	1.00	0.79	0.59	0.52	0.65
imatinib-dose-adjust	0.61	0.16	0.90	0.28	0.76	0.68	0.84
avapritinib-sensitive	0.08	0.08	1.00	0.15	0.54	0.45	0.65

Supplementary Table 6. Performance of the ML models for predicting treatment sensitivity categories in GIST. For each category, the table reports accuracy, precision, recall, F1 score, and the AUC with 95% CI.

medRxiv preprint doi: https://doi.org/10.64898/2026.02.02.26345350; this version posted February 3, 2026. The copyright holder for this preprint (which was not certified by peer review) is the author/funder, who has granted medRxiv a license to display the preprint in perpetuity.
It is made available under a CC-BY 4.0 International license .

Characteristics	All C1 patients	Training C1 patients	Internal Validation C1 patients	External Validation C1 patients	Training vs. int. Valid. p-value	Training vs. ext. valid p-value
**Age (continuous. years)**	64.2 [55 - 72.4] (11.8 - 97.9)	64.2 [55 - 72.4] (11.8 - 97.9)	64.9 [55 - 71.9] (17.7 - 91.3)	62 [51.7 - 72] (12 - 93.9)	0.9162	**0.0439***
**Age groups**						
≤ 64.2 years	1154/2285 (50.5)	720/1436 (50.1)	172/361 (47.6)	262/488 (53.7)	0.4305	0.1927
> 64.2 years	1131/2285 (49.5)	716/1436 (49.9)	189/361 (52.4)	226/488 (46.3)		
**Sex**						
Male	1170/2285 (51.2)	758/1436 (52.8)	164/361 (45.4)	248/488 (50.8)	**0.0147***	0.4848
Female	1115/2285 (48.8)	678/1436 (47.2)	197/361 (54.6)	240/488 (49.2)		
**Tumor site (detailed)**						
colorectal	76/2285 (3.3)	47/1436 (3.3)	15/361 (4.2)	14/488 (2.9)	0.7147	0.1188
small bowell	724/2285 (31.7)	437/1436 (30.4)	114/361 (31.6)	173/488 (35.5)		
oesophagus	17/2285 (0.7)	11/1436 (0.8)	3/361 (0.8)	3/488 (0.6)		
other	36/2285 (1.6)	18/1436 (1.3)	7/361 (1.9)	11/488 (2.3)		
stomach	1432/2285 (62.7)	923/1436 (64.3)	222/361 (61.5)	287/488 (58.8)		
**Tumor site (binary)**						
Other	853/2285 (37.3)	513/1436 (35.7)	139/361 (38.5)	201/488 (41.2)	0.3572	**0.0353***
Stomach	1432/2285 (62.7)	923/1436 (64.3)	222/361 (61.5)	287/488 (58.8)		
**Tumor size (continuous. mm)**	35 [8 - 70] (0.1 - 450)	35 [8 - 70] (0.1 - 450)	30 [6.5 - 70] (0.1 - 310)	55 [35 - 90] (1 - 340)	0.1257	**<0.0001*****
**Tumor size groups**						
≤ 5 cm	1373/2285 (60.1)	902/1436 (62.8)	238/361 (65.9)	233/488 (47.7)	0.5124	**<0.0001*****
> 5 and ≤ 10 cm	576/2285 (25.2)	329/1436 (22.9)	78/361 (21.6)	169/488 (34.6)		
> 10 cm	336/2285 (14.7)	205/1436 (14.3)	45/361 (12.5)	86/488 (17.6)		
**Mitotic index (continuous)**	3 [1 - 10] (0 - 300)	3 [1 - 10] (0 - 300)	3 [1 - 10] (0 - 248)	3 [1.8 - 7] (0 - 175)	0.7166	0.4805
**Mitotic index groups**						
≤ 5	1457/2285 (63.8)	899/1436 (62.6)	231/361 (64)	327/488 (67)	0.4824	**0.0034****
> 5 and ≤ 10	332/2285 (14.5)	206/1436 (14.3)	43/361 (11.9)	83/488 (17)		
> 10	496/2285 (21.7)	331/1436 (23.1)	87/361 (24.1)	78/488 (16)		
**Tumor rupture**						

medRxiv preprint doi: https://doi.org/10.64898/2026.02.02.26345350; this version posted February 3, 2026. The copyright holder for this preprint (which was not certified by peer review) is the author/funder, who has granted medRxiv a license to display the preprint in perpetuity.
It is made available under a CC-BY 4.0 International license .

No	2023/2183 (92.7)	1283/1385 (92.6)	316/352 (89.8)	424/446 (95.1)	0.0963	0.0951
Yes	160/2183 (7.3)	102/1385 (7.4)	36/352 (10.2)	22/446 (4.9)		
**Miettinen score**						
Low risk	1017/2285 (44.5)	606/1436 (42.2)	158/361 (43.8)	253/488 (51.8)	0.8099	**0.0007*** **
Intermediate risk	519/2285 (22.7)	347/1436 (24.2)	82/361 (22.7)	90/488 (18.4)		
High risk	749/2285 (32.8)	483/1436 (33.6)	121/361 (33.5)	145/488 (29.7)		
**Miettinen-Joensuu AFIP criteria**						
Low risk	993/2285 (43.5)	589/1436 (41)	151/361 (41.8)	253/488 (51.8)	0.6052	**0.0002*** **
Intermediate risk	485/2285 (21.2)	325/1436 (22.6)	73/361 (20.2)	87/488 (17.8)		
High risk	807/2285 (35.3)	522/1436 (36.4)	137/361 (38)	148/488 (30.3)		
**Mutational status**						
Favorable	1144/1963 (58.3)	771/1322 (58.3)	173/326 (53.1)	200/315 (63.5)	0.0980	0.1063
Unfavorable	819/1963 (41.7)	551/1322 (41.7)	153/326 (46.9)	115/315 (36.5)		
**DL Score C1 (continuous)**	-0.5 [-1.3 - 0.3] (-3.2 - 2.5)	-0.5 [-1.3 - 0.3] (-3.2 - 2.5)	-0.6 [-1.4 - 0.2] (-2.7 - 2.5)	-0.5 [-1.3 - 0.2] (-2.9 - 2.3)	0.6332	0.9192
**DL Score C1 (binary)**						
≤ Median score in Training C1	1151/2285 (50.4)	718/1436 (50)	190/361 (52.6)	243/488 (49.8)	0.4037	0.9794
> Median score	1134/2285 (49.6)	718/1436 (50)	171/361 (47.4)	245/488 (50.2)		
**Adjuvant TKI therapy**						
No	1491/2285 (65.3)	889/1436 (61.9)	207/361 (57.3)	395/488 (80.9)	0.1260	**<0.0001*** **
Yes	794/2285 (34.7)	547/1436 (38.1)	154/361 (42.7)	93/488 (19.1)		

Supplementary Table 7. Baseline characteristics of patients from the C1 cohort included in the recurrence-free survival (RFS) analysis. Data are presented as numbers with percentages in parentheses for categorical variables and as medians with interquartile and minimum–maximum ranges for continuous variables. Comparisons of baseline characteristics were performed between the training and internal validation (int. val.) sets and between the training and external validation (ext. val.) sets using Chi-square or Mann–Whitney tests, as appropriate. *p < 0.05; **p < 0.005; ***p < 0.001.

medRxiv preprint doi: https://doi.org/10.64898/2026.02.02.26345350; this version posted February 3, 2026. The copyright holder for this preprint (which was not certified by peer review) is the author/funder, who has granted medRxiv a license to display the preprint in perpetuity.
It is made available under a CC-BY 4.0 International license .

Characteristics	Cohort C1		Cohort C2		Cohort C3	
	**univariable HR (95% CI)**	**p-value**	**univariable HR (95% CI)**	**p-value**	**univariable HR (95% CI)**	**p-value**
**Age**						
≤ Median age in Training C1	REF	REF	REF	REF	REF	REF
> Median age	1 (0.81 - 1.23)	0.979	0.89 (0.66 - 1.19)	0.4211	1.1 (0.82 - 1.47)	0.5367
**Sex**						
Male	REF	REF	REF	REF	REF	REF
Female	0.78 (0.64 - 0.97)	**0.0233***	1.02 (0.76 - 1.37)	0.8855	0.76 (0.57 - 1.02)	0.0684
**Tumor site**						
Other	REF	REF	REF	REF	REF	REF
Stomach	0.52 (0.42 - 0.64)	**<0.0001*****	0.43 (0.32 - 0.58)	**<0.0001*****	0.57 (0.43 - 0.77)	**0.0002*****
**Tumor size**						
≤ 5 cm	REF	REF	REF	REF	REF	REF
> 5 and ≤ 10 cm	1.33 (1.03 - 1.73)	**0.0298***	2.16 (1.52 - 3.09)	**<0.0001*****	0.76 (0.51 - 1.15)	0.1943
> 10 cm	2.73 (2.13 - 3.51)	**<0.0001*****	5.69 (3.98 - 8.15)	**<0.0001*****	1.06 (0.74 - 1.52)	0.7600
**Mitotic index**						
≤ 5	REF	REF	REF	REF	REF	REF
> 5 and ≤ 10	2.82 (2.06 - 3.84)	**<0.0001*****	3.96 (2.56 - 6.11)	**<0.0001*****	1.62 (1.04 - 2.51)	**0.0334***
> 10	6.29 (4.96 - 7.97)	**<0.0001*****	13.92 (10.02 - 19.34)	**<0.0001*****	2.71 (1.93 - 3.79)	**<0.0001*****
**Tumor rupture**						
No	REF	REF	REF	REF	REF	REF
Yes	2.3 (1.68 - 3.15)	**<0.0001*****	3.65 (2.2 - 6.04)	**<0.0001*****	1.74 (1.19 - 2.52)	**0.0040****
**Miettinen-Joensuu AFIP criteria**						
Low risk	REF	REF	REF	REF		
Intermediate risk	3.96 (2.54 - 6.19)	**<0.0001*****	5.87 (3.51 - 9.81)	**<0.0001*****	1.42 (0.65 - 3.1)	0.3845
High risk	12.88 (8.72 - 19)	**<0.0001*****	22.74 (14.51 - 35.63)	**<0.0001*****	4.42 (2.17 - 9.01)	**<0.0001*****
**DL Scores Ci**						
≤ Median score in Train Ci	REF	REF	REF	REF	REF	REF
> Median score	8.44 (6.14 - 11.61)	**<0.0001*****	9.44 (5.87 - 15.2)	**<0.0001*****	4.74 (3.34 - 6.74)	**<0.0001*****

medRxiv preprint doi: https://doi.org/10.64898/2026.02.02.26345350; this version posted February 3, 2026. The copyright holder for this preprint (which was not certified by peer review) is the author/funder, who has granted medRxiv a license to display the preprint in perpetuity.
It is made available under a CC-BY 4.0 International license .

**Mutation group**						
Favorable	REF	REF	REF	REF	REF	REF
Unfavorable	2.74 (2.19 - 3.42)	**<0.0001*******	3.03 (2.23 - 4.12)	**<0.0001*******	1.97 (1.43 - 2.72)	**<0.0001*******
**Adjuvant TKI therapy**						
No	REF	REF	-	-	-	-
Yes	1.48 (1.2 - 1.82)	**0.0002*******	-	-	-	-
**Duration of adjuvant TKI**						
< 12 months	-	-	-	-	REF	REF
12-36 months	-	-	-	-	0.6 (0.42 - 0.85)	**0.0040******
> 36 months	-	-	-	-	0.34 (0.22 - 0.51)	**<0.0001*******

Supplementary Table 8. Univariable recurrence-free survival (RFS) analysis in the Training datasets from the C1, C2 and C3 cohorts. Other abbreviations: CI: confidence interval, DL: deep learning, REF: level of reference for univariable Cox regression, TKI: tyrosine kinase inhibitor. *: p < 0.05; **: p < 0.005; ***: p < 0.001.

Cohort	Model	Performances in Training§		Performances in int. val.		Performances in ext. val.	
		C-index (95% CI)	IBS (95% CI)	C-index (95% CI)	IBS (95% CI)	C-index (95% CI)	IBS (95% CI)
C1	**Mitotic model**	0.68 (0.626-0.734)	0.13 (0.125-0.136)	0.678 (0.629-0.739) (***)	0.134 (0.117-0.155) (***)	0.663 (0.597-0.731) (ns)	0.158 (0.136-0.187) (ns)
	**Simple DL model**	0.804 (0.765-0.844)	**0.111 (0.093-0.129)**	0.79 (0.739-0.847)	0.11 (0.09-0.13)	0.67 (0.589-0.752)	0.15 (0.121-0.181)
	**Pathological M.J. model**	0.774 (0.71-0.838)	0.116 (0.102-0.13)	0.797 (0.753-0.843) (ns)	0.108 (0.09-0.126) (ns)	**0.797 (0.744-0.876) (ns)**	**0.122 (0.096-0.15) (ns)**
	**Continuous M.J. model**	0.725 (0.667-0.783)	0.129 (0.121-0.137)	0.713 (0.664-0.774) (**)	0.126 (0.11-0.148) (**)	0.735 (0.673-0.81) (ns)	0.137 (0.118-0.162) (ns)
	**Deep M.J. model**	**0.813 (0.768-0.858)**	0.112 (0.094-0.131)	**0.798 (0.753-0.852)**	**0.107 (0.088-0.127)**	0.699 (0.627-0.774)	0.145 (0.115-0.177)
C2	**Mitotic model**	0.74 (0.678-0.802)	0.118 (0.098-0.138)	0.763 (0.679-0.862) (*)	0.111 (0.082-0.138) (***)	0.722 (0.641-0.822) (ns)	0.13 (0.105-0.164) (ns)
	**Simple DL model**	0.828 (0.787-0.869)	0.099 (0.083-0.116)	0.861 (0.794-0.929)	0.075 (0.057-0.099)	0.682 (0.586-0.764)	0.138 (0.097-0.171)
	**Pathologi**	0.833 (0.794-	**0.095 (0.076-**	0.862 (0.816-	0.092 (0.064-	**0.825 (0.757-**	**0.099 (0.066-**

medRxiv preprint doi: https://doi.org/10.64898/2026.02.02.26345350; this version posted February 3, 2026. The copyright holder for this preprint (which was not certified by peer review) is the author/funder, who has granted medRxiv a license to display the preprint in perpetuity.
It is made available under a CC-BY 4.0 International license .

	**cal M.J. model**	0.873)	**0.113)**	0.918) (ns)	0.116) (ns)	**0.892) (ns)**	**0.129) (**)**
	**Continuous M.J. model**	0.794 (0.707-0.88)	0.112 (0.089-0.135)	0.827 (0.763-0.89) (ns)	0.101 (0.076-0.129) (ns)	0.798 (0.718-0.891) (ns)	0.105 (0.08-0.129) (**)
	**Deep M.J. model**	**0.845 (0.809-0.881)**	0.103 (0.088-0.117)	**0.870 (0.807-0.933)**	**0.075 (0.051-0.102)**	0.739 (0.641-0.822)	0.131 (0.094-0.162)
**C3**	**Mitotic model**	0.595 (0.536-0.653)	0.144 (0.133-0.155)	0.714 (0.629-0.791) (ns)	0.139 (0.114-0.167) (*)	0.593 (0.461-0.724) (ns)	0.174 (0.124-0.217) (ns)
	**Simple DL model**	**0.729 (0.669-0.788)**	**0.124 (0.11-0.138)**	**0.798 (0.749-0.857)**	**0.114 (0.093-0.136)**	**0.599 (0.437-0.746)**	0.174 (0.135-0.221)
	**Pathological M.J. model**	0.651 (0.584-0.718)	0.139 (0.124-0.155)	0.74 (0.666-0.831) (ns)	0.136 (0.109-0.158) (ns)	0.558 (0.412-0.715) (ns)	**0.167 (0.123-0.207) (***)**
	**Continuous M.J. model**	0.628 (0.542-0.715)	0.142 (0.134-0.15)	0.692 (0.594-0.788) (*)	0.147 (0.121-0.178) (***)	0.441 (0.328-0.538) (ns)	0.188 (0.145-0.229) (ns)
	**Deep M.J. model**	0.734 (0.676-0.792)	**0.124 (0.113-0.136)**	0.792 (0.731-0.846)	**0.115 (0.092-0.141)**	0.54 (0.377-0.664)	0.190 (0.153-0.234)

Supplementary Table 9. Prognostic performances of survival models across cohorts C1–C3. Concordance indices (C-index) and integrated Brier scores (IBS) with 95% confidence intervals (CIs) are reported for the mitotic index–based model (“Mitotic”), the simple deep learning (DL) score model (“Simple DL”), the pathological Miettinen–Joensuu model (“Path. MJ”), the continuous Miettinen–Joensuu model (“Cont. MJ”), and the deep Miettinen–Joensuu model (“Deep MJ”) in training (out-of-fold cross-validation), internal validation, and external validation datasets. Results are shown for the full C1 cohort, the C2 subcohort (patients without adjuvant TKI therapy), and the C3 subcohort (patients with adjuvant TKI therapy). Significance levels from permutation tests comparing paired models are indicated as follows: ns, not significant; *p < 0.05; **p < 0.005; ***p < 0.001.

**Characteristics Cox Multivariable modeling**	**Cohort C1**		**Cohort C2**		**Cohort C3**	
	**HR (95% CI)**	**p-value**	**HR (95% CI)**	**p-value**	**HR (95% CI)**	**p-value**
DL Score (continuous)	2.83 (2.47-3.25)	**<0.0001*****	2.64 (2.23-3.13)	**<0.0001*****	3.09 (2.4-3.96)	**<0.0001*****
Mitotic index (continuous)	1 (1-1)	0.1750	1 (1-1.01)	**0.0165***	1 (1-1.01)	0.3221
Tumor size	1 (1-1)	**0.0030****	1 (1-1.01)	**0.0004*****	1 (1-1)	0.2627

medRxiv preprint doi: https://doi.org/10.64898/2026.02.02.26345350; this version posted February 3, 2026. The copyright holder for this preprint (which was not certified by peer review) is the author/funder, who has granted medRxiv a license to display the preprint in perpetuity.
It is made available under a CC-BY 4.0 International license .

(continuous)						
Tumor site (stomach)	0.54 (0.43-0.68)	**<0.0001*** **	0.45 (0.32-0.63)	**<0.0001*** **	0.55 (0.4-0.76)	**0.0003*** **
Tumor rupture (yes)	0.92 (0.66-1.29)	0.6468	1.27 (0.75-2.17)	0.3738	1.24 (0.83-1.85)	0.2918
Age (>median age)	1.39 (1.11-1.74)	**0.0044** **	1.22 (0.88-1.69)	0.2346	1.45 (1.06-1.98)	**0.0208* **
Sex (Female)	0.78 (0.62-0.98)	**0.0300* **	0.79 (0.58-1.1)	0.1610	0.94 (0.69-1.29)	0.7141
mutation (unfavorable)	1.99 (1.56-2.54)	**<0.0001*** **	1.89 (1.32-2.7)	**0.0005*** **	1.79 (1.27-2.52)	**0.0008*** **
Adjuvant TKI (yes)	0.55 (0.43-0.7)	**<0.0001*** **	**-**	**-**	**-**	**-**

Supplementary Table 10. Multivariable Cox regression analyses in cohorts C1–C3. Hazard ratios (HRs) with 95% confidence intervals (CIs) and p-values are reported for the deep learning (DL) score (continuous), mitotic index (continuous), tumor size (continuous), tumor site (stomach vs. other), tumor rupture, age (>median), sex (female vs. male), mutation status (unfavorable vs. favorable), and adjuvant TKI therapy (yes vs. no). Results are shown for the overall C1 cohort, the C2 subcohort (patients without adjuvant TKI therapy), and the C3 subcohort (patients with adjuvant TKI therapy). The DL score remained an independent prognostic factor in all cohorts, with hazard ratios ranging from 2.64 to 3.09. Other abbreviations: TKI, tyrosine kinase inhibitor. *p < 0.05; **p < 0.005; ***p < 0.001. Significant results are in bold.

**Characteristics**	**All C2 patients**	**Training C2 patients**	**Internal Validation C2 patients**	**External Validation C2 patients**	**Training vs. int. valid p-value**	**Training vs. ext. valid p-value**
**Age (continuous. years)**	64.9 [54.7 - 73.3] (11.8 - 97.9)	65 [55.5 - 73.7] (11.8 - 97.9)	66.7 [56 - 74.8] (17.2 - 93.1)	62 [51.5 - 72.2] (18 - 93.9)	0.1833	**0.0074***
**Age groups**						
≤ 64.2 years	717/1491 (48.1)	419/885 (47.3)	88/211 (41.7)	210/395 (53.2)	0.1617	0.0624
> 64.2 years	774/1491 (51.9)	466/885 (52.7)	123/211 (58.3)	185/395 (46.8)		
**Sex**						
Male	752/1491 (50.4)	439/885 (49.6)	110/211 (52.1)	203/395 (51.4)	0.5596	0.5958
Female	739/1491 (49.6)	446/885 (50.4)	101/211 (47.9)	192/395 (48.6)		
**Tumor site (detailed)**						
colorectal	45/1491 (3)	28/885 (3.2)	5/211 (2.4)	12/395 (3)	0.1640	0.2100
small bowell	408/1491 (27.4)	235/885 (26.6)	46/211 (21.8)	127/395 (32.2)		
oesophagus	10/1491 (0.7)	5/885 (0.6)	3/211 (1.4)	2/395 (0.5)		
other	16/1491 (1.1)	9/885 (1)	0/211 (0)	7/395 (1.8)		

medRxiv preprint doi: https://doi.org/10.64898/2026.02.02.26345350; this version posted February 3, 2026. The copyright holder for this preprint (which was not certified by peer review) is the author/funder, who has granted medRxiv a license to display the preprint in perpetuity.
It is made available under a CC-BY 4.0 International license .

stomach	1012/1491 (67.9)	608/885 (68.7)	157/211 (74.4)	247/395 (62.5)		
**Tumor site (binary)**						
Other	479/1491 (32.1)	277/885 (31.3)	54/211 (25.6)	148/395 (37.5)	0.1238	**0.0357***
Stomach	1012/1491 (67.9)	608/885 (68.7)	157/211 (74.4)	247/395 (62.5)		
**Tumor size (mm)**	40 [20 - 70] (0.1 - 400)	40 [10 - 65] (0.1 - 400)	37 [7.2 - 63] (0.2 - 240)	49 [30 - 75] (1 - 340)	0.4617	**<0.0001*** **
**Tumor size groups**						
≤ 5 cm	929/1491 (62.3)	568/885 (64.2)	139/211 (65.9)	222/395 (56.2)	0.8925	**0.0250***
> 5 and ≤ 10 cm	387/1491 (26)	218/885 (24.6)	49/211 (23.2)	120/395 (30.4)		
> 10 cm	175/1491 (11.7)	99/885 (11.2)	23/211 (10.9)	53/395 (13.4)		
**Mitotic index (continuous)**	3 [1 - 5] (0 - 300)	2 [1 - 5] (0 - 300)	3 [1 - 5] (0 - 250)	3 [1 - 5] (0 - 112)	0.3932	0.4203
**Mitotic index groups**						
≤ 5	1139/1491 (76.4)	675/885 (76.3)	159/211 (75.4)	305/395 (77.2)	0.9546	0.1328
> 5 and ≤ 10	171/1491 (11.5)	95/885 (10.7)	24/211 (11.4)	52/395 (13.2)		
> 10	181/1491 (12.1)	115/885 (13)	28/211 (13.3)	38/395 (9.6)		
**Tumor rupture**						
No	1361/1407 (96.7)	822/854 (96.3)	194/198 (98)	345/355 (97.2)	0.3235	0.5274
Yes	46/1407 (3.3)	32/854 (3.7)	4/198 (2)	10/355 (2.8)		
**Miettinen score**						
Low risk	927/1491 (62.2)	547/885 (61.8)	131/211 (62.1)	249/395 (63)	0.9603	0.8252
Intermediate risk	294/1491 (19.7)	179/885 (20.2)	41/211 (19.4)	74/395 (18.7)		
High risk	270/1491 (18.1)	159/885 (18)	39/211 (18.5)	72/395 (18.2)		
**Miettinen-Joensuu AFIP criteria**						
low	920/1491 (61.7)	542/885 (61.2)	129/211 (61.1)	249/395 (63)	0.9829	0.8234
intermediate	278/1491 (18.6)	167/885 (18.9)	39/211 (18.5)	72/395 (18.2)		
high	293/1491 (19.7)	176/885 (19.9)	43/211 (20.4)	74/395 (18.7)		
**Mutational status**						
Favorable	752/1491 (50.4)	526/783 (67.2)	126/184 (68.5)	176/246 (71.5)	0.8015	0.2283
Unfavorable	739/1491 (49.6)	257/783 (32.8)	58/184 (31.5)	70/246 (28.5)		
**DL Score C2 (continuous)**	-0.2 [-1 - 0.6] (-2.4 - 3.5)	-0.3 [-1.1 - 0.5] (-2.4 - 3.5)	-0.2 [-1 - 0.8] (-2.3 - 3.5)	0 [-0.7 - 0.7] (-2.2 - 3.3)	0.2260	**<0.0001*** **
**DL Score C2 (binary)**						
≤ Median score in Training C2	693/1491 (46.5)	443/885 (50.1)	100/211 (47.4)	150/395 (38)	0.5361	**<0.0001*** **
> Median score	798/1491 (53.5)	442/885 (49.9)	111/211 (52.6)	245/395 (62)		

medRxiv preprint doi: https://doi.org/10.64898/2026.02.02.26345350; this version posted February 3, 2026. The copyright holder for this preprint (which was not certified by peer review) is the author/funder, who has granted medRxiv a license to display the preprint in perpetuity.
It is made available under a CC-BY 4.0 International license .

Supplementary Table 11. Baseline characteristics of patients from the C2 cohort included in the recurrence-free survival (RFS) analysis. Data are presented as numbers with percentages in parentheses for categorical variables and as medians with interquartile and minimum–maximum ranges for continuous variables. Comparisons of baseline characteristics were performed between the training and internal validation (int. val.) sets and between the training and external validation (ext. val.) sets using Chi-square or Mann–Whitney tests, as appropriate. *p < 0.05; **p < 0.005; ***p < 0.001.

Characteristics	All C3 patients	Training C3 patients	Internal Validation C3 patients	External Validation C3 patients	Training vs. int. valid p-value	Training vs. ext. valid p-value
**Age (continuous, years)**	63 [53.1 - 70] (12 - 89.8)	63.8 [54 - 70.9] (15.4 - 89.8)	62 [50 - 68.8] (21.5 - 83)	62.2 [52.4 - 70.7] (12 - 85.9)	**0.0342***	0.7717
**Age groups**						
≤ 64.2 years	437/794 (55)	305/562 (54.3)	80/139 (57.6)	52/93 (55.9)	0.5476	0.8552
> 64.2 years	357/794 (45)	257/562 (45.7)	59/139 (42.4)	41/93 (44.1)		
**Sex**						
Male	418/794 (52.6)	301/562 (53.6)	72/139 (51.8)	45/93 (48.4)	0.7814	0.4160
Female	376/794 (47.4)	261/562 (46.4)	67/139 (48.2)	48/93 (51.6)		
**Tumor site (detailed)**						
colorectal	31/794 (3.9)	22/562 (3.9)	7/139 (5)	2/93 (2.2)	0.9461	0.1527
small bowell	316/794 (39.8)	216/562 (38.4)	54/139 (38.8)	46/93 (49.5)		
oesophagus	7/794 (0.9)	5/562 (0.9)	1/139 (0.7)	1/93 (1.1)		
other	20/794 (2.5)	12/562 (2.1)	4/139 (2.9)	4/93 (4.3)		
stomach	420/794 (52.9)	307/562 (54.6)	73/139 (52.5)	40/93 (43)		
**Tumor site**						
Other	374/794 (47.1)	255/562 (45.4)	66/139 (47.5)	53/93 (57)	0.7251	0.0492*
Stomach	420/794 (52.9)	307/562 (54.6)	73/139 (52.5)	40/93 (43)		
**Tumor size (mm)**	25 [7.5 - 90] (0.8 - 450)	14 [7 - 80] (0.8 - 350)	15 [7 - 70] (2.1 - 450)	90 [64 - 130] (19 - 300)	0.6775	**<0.0001*** **
**Tumor size groups**						
≤ 5 cm	444/794 (55.9)	346/562 (61.6)	87/139 (62.6)	11/93 (11.8)	**0.0039** **	**<0.0001*** **
> 5 and ≤ 10 cm	189/794 (23.8)	102/562 (18.1)	38/139 (27.3)	49/93 (52.7)		
> 10 cm	161/794 (20.3)	114/562 (20.3)	14/139 (10.1)	33/93 (35.5)		
**Mitotic index (continuous)**	7.5 [2.2 - 20.8] (0 - 300)	7 [2 - 20] (0 - 292)	7 [2 - 23.5] (0 - 300)	9 [6 - 21] (0 - 175)	0.6084	**0.0065***
**Mitotic index groups**						
≤ 5	318/794 (40.1)	241/562 (42.9)	55/139 (39.6)	22/93 (23.7)	0.5602	**0.0002*** **
> 5 and ≤ 10	161/794 (20.3)	100/562 (17.8)	30/139 (21.6)	31/93 (33.3)		

medRxiv preprint doi: https://doi.org/10.64898/2026.02.02.26345350; this version posted February 3, 2026. The copyright holder for this preprint (which was not certified by peer review) is the author/funder, who has granted medRxiv a license to display the preprint in perpetuity.
It is made available under a CC-BY 4.0 International license .

> 10	315/794 (39.7)	221/562 (39.3)	54/139 (38.8)	40/93 (43)		
**Tumor rupture**						
No	662/776 (85.3)	468/550 (85.1)	115/135 (85.2)	79/91 (86.8)	>0.9999	0.7870
Yes	114/776 (14.7)	82/550 (14.9)	20/135 (14.8)	12/91 (13.2)		
**Miettinen score**						
Low risk	90/794 (11.3)	68/562 (12.1)	18/139 (12.9)	4/93 (4.3)	0.9464	**0.0008*** **
Intermediate risk	225/794 (28.3)	167/562 (29.7)	42/139 (30.2)	16/93 (17.2)		
High risk	479/794 (60.3)	327/562 (58.2)	79/139 (56.8)	73/93 (78.5)		
**Miettinen-Joensuu AFIP criteria**						
Low risk	73/794 (9.2)	52/562 (9.3)	17/139 (12.2)	4/93 (4.3)	0.4679	**0.0108***
Intermediate risk	207/794 (26.1)	152/562 (27)	40/139 (28.8)	15/93 (16.1)		
High risk	514/794 (64.7)	358/562 (63.7)	82/139 (59)	74/93 (79.6)		
**Mutational status**						
Favorable	316/750 (42.1)	235/545 (43.1)	57/136 (41.9)	24/69 (34.8)	0.8747	0.2334
Unfavorable	434/750 (57.9)	310/545 (56.9)	79/136 (58.1)	45/69 (65.2)		
**DL Score C3 (continuous)**	0 [-0.6 - 0.5] (-2.1 - 1.8)	-0.1 [-0.6 - 0.4] (-2.1 - 1.8)	0 [-0.7 - 0.4] (-1.7 - 1.4)	0.3 [-0.6 - 0.7] (-1.7 - 1.4)	0.8978	**0.0163***
**DL Score C3 (binary)**						
≤ Median score in Training C3	386/794 (48.6)	281/562 (50)	69/139 (49.6)	36/93 (38.7)	>0.9999	0.0566
> Median score	408/794 (51.4)	281/562 (50)	70/139 (50.4)	57/93 (61.3)		
**Duration of adjuvant TKI**						
< 12 months	149/783 (19)	110/557 (19.7)	31/138 (22.5)	8/88 (9.1)	0.7747	**<0.0001*** **
12-36 months	412/783 (52.6)	278/557 (49.9)	67/138 (48.6)	67/88 (76.1)		
> 36 months	222/783 (28.4)	169/557 (30.3)	40/138 (29)	13/88 (14.8)		

Supplementary Table 12. Baseline characteristics of patients from the C3 cohort included in the recurrence-free survival (RFS) analysis. Data are presented as numbers with percentages in parentheses for categorical variables and as medians with interquartile and minimum–maximum ranges for continuous variables. Comparisons of baseline characteristics were performed between the training and internal validation (int. val.) sets and between the training and external validation (ext. val.) sets using Chi-square or Mann–Whitney tests, as appropriate. *p < 0.05; **p < 0.005; ***p < 0.001.

medRxiv preprint doi: https://doi.org/10.64898/2026.02.02.26345350; this version posted February 3, 2026. The copyright holder for this preprint (which was not certified by peer review) is the author/funder, who has granted medRxiv a license to display the preprint in perpetuity.
It is made available under a CC-BY 4.0 International license .

Outcome	Characteristics	Survival probability at 5 years	log-rank p-value	HR (95% CI)	Cox p-value
RFS	**Deep MJ C2 groups**				
	≤ median	43.77 (33.72 - 56.82)	**0.0007*** **	REF	REF
	> median	23.12 (15.42 - 34.66)		1.86 (1.29 - 2.67)	**0.0008*** **
	**Deep MJ C3 groups**				
	≤ median	78.96 (73.3 - 85.05)	**<0.0001*** **	REF	REF
	> median	51.03 (44.5 - 58.52)		3.44 (2.5 - 4.72)	**<0.0001*** **
	**Summary deep MJ C2 and C3 groups**				
	C2 - low risk Deep MJ C2	43.77 (33.72 - 56.82)	**<0.0001*** **	REF	REF
	C2 - high risk Deep MJ C2	23.12 (15.42 - 34.66)		0.6 (0.47 - 0.75)	**<0.0001*** **
	C3 - low_risk Deep MJ C3	78.96 (73.3 - 85.05)		1.29 (1.02 - 1.65)	**0.0352***
	C3 - high risk Deep MJ C3	51.03 (44.5 - 58.52)		3.59 (2.8 - 4.59)	**<0.0001*** **
OS	**Deep MJ C2 groups**				
	≤ median	88.69 (81.46 - 96.56)	**0.0333***	REF	REF
	> median	76.94 (68.07 - 86.95)		1.78 (1.04 - 3.06)	**0.0356***
	**Deep MJ C3 groups**				
	≤ median	95.13 (92.22 - 98.12)	**<0.0001*** **	REF	REF
	> median	84.78 (79.87 - 89.99)		3.44 (2.04 - 5.79)	**<0.0001*** **
	**Summary deep MJ C2 and C3 groups**				
	C2 - low risk Deep MJ C2	88.69 (81.46 - 96.56)	**<0.0001*** **	REF	REF
	C2 - high risk Deep MJ C2	76.94 (68.07 - 86.95)		0.85 (0.59 - 1.23)	0.3987
	C3 - low_risk Deep MJ C3	95.13 (92.22 - 98.12)		1.39 (0.96 - 2.02)	0.085
	C3 - high risk Deep MJ C3	84.78 (79.87 - 89.99)		2.99 (2.03 - 4.39)	**<0.0001*** **

medRxiv preprint doi: https://doi.org/10.64898/2026.02.02.26345350; this version posted February 3, 2026. The copyright holder for this preprint (which was not certified by peer review) is the author/funder, who has granted medRxiv a license to display the preprint in perpetuity.
It is made available under a CC-BY 4.0 International license .

Supplementary Table 13. Recurrence-free survival (RFS) and overall survival (OS) in patients from C2 and C3 classified as high risk according to the pathological Miettinen–Joensuu (MJ) scoring system and carrying imatinib-sensitive mutations.

Hazard ratios (HRs) with 95% confidence intervals (CIs) are shown; REF indicates the reference category. *p < 0.05; **p < 0.005; ***p < 0.001. Significant results are in bold.
